# Editorial: Atrial fibrillation: insights on mechanisms, mapping and catheter ablation

**DOI:** 10.3389/fcvm.2023.1280925

**Published:** 2023-09-27

**Authors:** Sergio Conti, Atul Verma, Andrea Natale, Claudio Tondo

**Affiliations:** ^1^ Department of Cardiac Electrophysiology, ARNAS Civico Di Cristina Benfratelli, Palermo, Italy; ^2^ Division of Cardiology, McGill University Health Centre, McGill University, Montreal, QC, Canada; ^3^ St. David’s Medical Center, Texas Cardiac Arrhythmia Institute, Austin, TX, United States; ^4^ Interventional Electrophysiology, Scripps Clinic, San Diego, CA, United States; ^5^ Department of Internal Medicine, Metro Health Medical Center, Case Western Reserve University School of Medicine, Cleveland, OH, United States; ^6^ Centro Cardiologico Monzino, IRCCS, Dipartimento di Scienze Biochimiche, Chirurgiche e Odontoiatriche, Università Degli Studi, Milano, Italy

**Keywords:** atrial fibrillation, catheter ablation, atrial fibrillation catheter ablation, atrial fibrillation management, atrial fibrillation mechanisms

**Editorial on the Research Topic** Atrial fibrillation: insights on mechanisms, mapping and catheter ablation

Atrial fibrillation (AF) is the most common type of cardiac arrhythmia and is associated with an increased risk of stroke, heart failure, and mortality (1). The number of patients affected is expected to grow continuously in the following years, owing to extended longevity in the general population and intensifying screening for undiagnosed AF (1). In recent years, significant progress has been made regarding identifying and treating risk factors, evaluating electrical and structural remodeling, preprocedural imaging, understanding electrophysiological mechanisms and substrates underlying AF, and ablation strategies and technologies. The main “players” widely recognized as the mechanisms underlying AF are the pulmonary veins (PVs) and extra-PVs triggers and their interaction with the left atrial substrate. A better understanding of these mechanisms has influenced AF's mapping and ablation strategies. In particular, technological and technical improvements of catheter ablation are highly impressive. Indeed, the continuous implementation of new technologies has made AF ablation faster, more effective, and safer. Within the last years, high-definition mapping technologies, additional improvements with novel point-by-point radiofrequency (RF), and single-shot devices have captured the interest of most of the scientific community. The most important innovation, however, seems to be the introduction of pulsed-field ablation (PFA) technology.

In addition, research is rich in new publications focusing on the timing of AF treatment. The findings of the EAST-AFNET 4 are giving further impulse to the electrophysiology community to propose a prompt treatment of atrial fibrillation (2). Since the late 90s, catheter ablation emerged as a promising treatment strategy for patients with AF. After the seminal work of Haissaguerre et al. (3) led to the development of pulmonary vein isolation (PVI), aiming at electrical disconnection of the PVs, nowadays catheter ablation is a well-established treatment for patients with symptomatic, drug-refractory AF. Recent guidelines clearly state that the cornerstone of any AF ablation procedure is the complete isolation of the PVs by linear lesions around their antrum, either using point-by-point RF ablation or single-shot ablation devices, irrespective of the AF type (1). However, there is still a lack of consensus regarding the ablation strategy to be adopted, especially in specific sets of patients.

Through contributions from leading experts in the field, the present Special Issue presents a contemporary perspective on AF mechanisms, mapping, and catheter ablation. In recent years, several studies focused on defining the substrate of the left atrium in patients with AF. The ERASE-AF trial recently showed that ablation of fibrotic tissue determined by mapping low-voltage areas improved the outcome in patients with persistent AF (4). In this Issue, Shao et al. evaluated the role of left atrial epicardial adipose tissue and low-voltage areas. Moreover, two reviews have been included in the Issue focused on how to deal with and manage atrial fibrotic tissue. In the RF field, after contact force sensing catheters, the introduction of more advanced lesion parameters, such as the Ablation Index (AI) and Lesion1 Index (LSI), allowed operators to perform ablation procedures more safely and efficiently. The reader of the Issue will find insights on the lesion durability of LSI-guided PVI and the risk factors for late reconnections of PVs in the paper of Mujovic et al.. Liu et al. compared the role of AI in guiding AF ablation using high-power vs. low-power settings. Beyond PVI, the optimal ablation strategy remains a matter of debate. The role of the vein of Marshall has been assessed in the VENUS randomized clinical trial and, more recently, in the prospective single-center Marshall-PLAN (5, 6). Langmuur et al. reported an accurate method to localize the ligament of Marshall by using unipolar electrograms in activation and voltage maps.

Finally, what can be a real game-changer in the treatment of AF, or at least it has been expected, is the introduction in the clinical practice of PFA. An increasing body of evidence confirms the more than promising outcomes of this new technology. The PULSED AF Pivotal trial reported that PFA was successful at treating AF at 12 months in 66.2% of patients with paroxysmal AF and 55.1% with persistent AF (7). In this Issue, Magni et al. reported their initial experience on a large population undergoing AF ablation using PFA.

There is no doubt that more has to come from ongoing and future research, particularly in the field of tailored approaches in persistent and long-standing persistent AF patients, imaging and substrate mapping integration, comparison between different technologies, and evaluation of long-term outcomes. In the meantime, we hope the Frontiers in Cardiovascular Medicine readers will find the current Special Issue interesting and helpful in broadening their knowledge of current state-of-the-art AF research.
